# Psychological risk factors and resources for low back pain intensity and back health in daily life: An ecological momentary assessment study

**DOI:** 10.1111/aphw.70080

**Published:** 2025-09-22

**Authors:** Karolina Kolodziejczak‐Krupp, Lea O. Wilhelm, Lotte‐Eleonora Diering, Valerie Zipper, Jana Maas, Thomas Schäfer, Matthias Pumberger, Hendrik Schmidt, Christoph Stein, Lena Fleig

**Affiliations:** ^1^ Department of Psychology MSB Medical School Berlin Berlin Germany; ^2^ Department of Education and Psychology Freie Universität Berlin Berlin Germany; ^3^ Institute of Medical Sociology Charité – Universitätsmedizin Berlin Berlin Germany; ^4^ Sana Hospital Lichtenberg Berlin Germany; ^5^ Department of Psychology HMU Health and Medical University Erfurt Erfurt Germany; ^6^ Center for Musculoskeletal Surgery Charité – Universitätsmedizin Berlin Berlin Germany; ^7^ Julius Wolff Institute Berlin Institute of Health at Charité – Universitätsmedizin Berlin Berlin Germany; ^8^ Department of Anaesthesiology and Intensive Care Medicine Charité – Universitätsmedizin Berlin, Campus Benjamin Franklin Berlin Germany

**Keywords:** ambulatory assessment, fear of movement, fear‐avoidance model, kinesiophobia, leisure‐time physical activity, pain self‐efficacy

## Abstract

Chronic low back pain (cLBP) is a highly prevalent and disabling health condition. Identifying risk factors and resources for low back pain (LBP) and back health in everyday life is crucial for its prevention and management. This study examined moment‐to‐moment fluctuations in pain intensity and perceived back health and their associations with fear of movement, pain self‐efficacy, and leisure‐time physical activity in individuals with (*n* = 128) and without (*n* = 94) cLBP. Data were collected five times per day for 14 days (*n*_measurements_ = 13,292). Participants with cLBP reported experiencing LBP in 45% of all measurements, with a mean intensity of 3.16 (range: 1–9), whereas participants without cLBP reported LBP in 6.9% of measurements (*M*_intensity_ = 2.38). Multilevel analyses including participants with and without cLBP showed that greater momentary fear of movement, lower momentary pain self‐efficacy, and higher momentary levels of leisure‐time physical activity were associated with higher pain intensity (*β* = .05, *p* = .042, *β* = −.15, *p* < .001, and *β* = .04, *p* = .001). Greater momentary fear of movement and lower momentary pain self‐efficacy were associated with poorer back health (*β* = −.07, *p* = .004 and *β* = .16, *p* < .001). The observed within‐person associations highlight the potential for ecological momentary interventions targeting modifiable psychological factors related to cLBP in daily life, particularly pain self‐efficacy.

## INTRODUCTION

Low back pain (LBP) is a highly prevalent and disabling health condition, with long‐term projections indicating a significant and continuing rise in the number of individuals affected (Ferreira et al., 2023). The substantial personal burden and healthcare costs associated with chronic LBP (cLBP) underscore the urgency of its prevention and management as a critical public health priority (Becker et al., 2010; Geurts et al., 2018). The biopsychosocial model of cLBP has emphasized its multicausality and, importantly, the need to address the psychological factors involved in cLBP (Waddell, 1987). Similarly, a growing body of empirical evidence (Benz et al., 2021; Nieminen et al., 2021; Taheri et al., 2025) and clinical practice guidelines (Chenot et al., 2017) have highlighted the crucial role of psychological factors in cLBP. However, to date, most research on psychosocial factors related to LBP has been cross‐sectional or longitudinal with relatively few measurement points (Levenig et al., 2024; Wertli et al., 2014). While such findings shed light on factors that differentiate well *between* individuals (e.g. depression; Nieminen et al., 2021; Pinheiro et al., 2016), other factors may show stronger short‐term fluctuations and covariation with LBP in daily life (i.e. *within* individuals). A thorough understanding of the real‐life dynamics of LBP is essential, particularly for the development of effective noninvasive and nonpharmacological interventions in daily life, paving the way for new approaches to the prevention and management of cLBP (De Melo Santana et al., 2023; Rintala et al., 2022). To this end, ecological momentary assessment (EMA) is particularly well suited to address questions of intraindividual variability and thus provide ecologically valid insights into cLBP (May et al., 2018).

Therefore, in this observational study, we utilize an EMA design to examine LBP and its psychological correlates in daily life. Our primary objective is to identify modifiable psychological risk factors and resources for LBP and back health. Additionally, we focus on physical activity as a key health behavior that may serve as a critical target for interventions in daily life.

### Fluctuations in pain intensity and back health in daily life

Temporal characteristics such as the duration and frequency of LBP (e.g. daily or almost daily occurrence of pain for at least 3 months; Freburger et al., 2009; Häuser et al., 2014; Neuhauser et al., 2005; von der Lippe et al., 2021) are defining aspects of cLBP. For example, the Graded Chronic Pain Scale‐Revised (GCPS‐R) proposed by Von Korff and colleagues (2020) assesses momentary, strongest, and average pain intensity over the past 3 months. While cross‐sectional measurements that capture phenomena at a single point in time offer efficiency, they fall short of capturing the nuanced, moment‐to‐moment experiences of pain in real‐life contexts. To illustrate, the commonly used clinical criterion for diagnosing cLBP as persisting *daily* or *almost daily* for the past 3 months lacks strong empirical support. Notably, single‐item measures of LBP in EMA studies have exhibited good to excellent validity in previous studies (Overton et al., 2023) and provide a practical approach for examining within‐person associations between pain and other variables (May et al., 2018).

Pain intensity is a critical indicator of LBP and a key patient‐reported outcome in clinical settings (Olsen et al., 2018; Pogatzki‐Zahn et al., 2019). However, effective prevention of cLBP may require additional assessments that capture fluctuations in back health before pain becomes chronic. A complementary approach to pain reporting is the assessment of back‐specific patient‐reported health indicators, such as back health (based on the salutogenic model; Antonovsky, 2024; Gruszka et al., 2019). Despite its potential relevance, empirical evidence on the utility of self‐reported measures of back health in individuals with cLBP is scarce.

### Psychological risk factors and resources for pain intensity and back health

The biopsychosocial model of pain implies that to understand the relationship between LBP and psychological factors, perspectives from different disciplines must be combined, for example, pain medicine, health, and pain psychology (Kolodziejczak‐Krupp et al., 2025; Wilhelm et al., 2025). One well‐established framework that combines such perspectives is the fear‐avoidance model of pain (Vlaeyen et al., 1995; Vlaeyen et al., 2016). This model suggests that perceiving pain as highly threatening leads to a fear response. In particular, *fear of movement*, along with the resulting avoidance behavior (e.g. low levels of physical activity), corresponds to the increased need to control pain. While this mechanism may be protective and thus adaptive in the case of acute injury or pain, in the long term, avoidance behavior interferes with relevant life activities. Furthermore, fear of movement is reinforced by avoidance behavior, which, once established, tends to persist and exacerbate pain, creating a self‐perpetuating vicious cycle (Vlaeyen et al., 2016). While substantial cross‐sectional, between‐person evidence supports the association between fear of movement and pain intensity (Markfelder & Pauli, 2020) recent research also highlights similar associations at the momentary, within‐person level for cLBP (Kichline et al., 2022).

In addition to pain‐related risk factors such as fear of movement, psychological resources, as outlined in the back health behavior model (BHBM), play a crucial role in the management of LBP (Kolodziejczak‐Krupp et al., 2025; Wilhelm et al., 2025). One key psychological factor is *pain self‐efficacy*, which refers to individuals' confidence in their ability to perform activities despite experiencing pain (Nicholas, 2007). This concept is rooted in Bandura's concept of self‐efficacy, which emphasizes the expectation of successfully coping with challenges (Bandura, 1986), but with the added difficulty of managing those challenges while in pain (Nicholas, 2007). Measurement of pain self‐efficacy has now become increasingly parsimonious, with validated short scales (e.g. four‐ or two‐item versions derived from the original 10‐item Pain Self‐Efficacy Questionnaire, PSEQ, Chiarotto et al., 2016; Nicholas et al., 2015) suggesting that a reduced number of items may still be a valid indicator of pain self‐efficacy. Cross‐sectional findings have highlighted that pain self‐efficacy is just as important a predictor of pain intensity as fear of movement (Ferrari et al., 2016). Daily life studies on self‐efficacy related to aspects other than pain (e.g. action self‐efficacy and coping self‐efficacy in psychology of behavior change, based on the Health Action Process Approach [HAPA]; Schwarzer & Renner, 2000), in turn, highlight the importance of self‐efficacy for health behavior change and thus possible daily life interventions (Haag et al., 2023).

### Health behavior and its links to pain intensity and back health

Applying the fear‐avoidance model of pain in the context of health behavior, fear of movement would result in avoidance behavior such as ceasing or reducing activity, particularly physical activity. Consistent with this, research has shown that fewer fear‐avoidance beliefs are associated with increased physical activity and lower incidence of LBP (Fujii et al., 2013). At the same time, clinical practice guidelines recommend increasing physical activity as a component of LBP management to reduce pain and prevent and reduce disability (Burton et al., 2006; Oliveira et al., 2018; Pfeifer et al., 2016). In particular, *leisure‐time physical activity* (LTPA) is considered beneficial for people with cLBP, with the underlying mechanism being related to both better physical and psychosocial functioning, as indicated by, for example, higher muscle strength, lower bone loss, better mood, lower social isolation, and higher quality of life (Pinto et al., 2014). A study by Gupta et al. (2022) showed that increasing moderate‐to‐vigorous physical activity at work was associated with a higher risk of long‐term sickness absence due to cLBP, whereas increasing moderate‐to‐vigorous physical activity during leisure time was associated with a lower risk of absence. Similarly, a systematic review showed that higher levels of LTPA were associated with a lower risk of cLBP compared to those with no physical activity (Shiri & Falah‐Hassani, 2017). Although there is evidence for cross‐sectional (Fjeld et al., 2023) and longitudinal (Pinto et al., 2014) associations between self‐reported LTPA and LBP, it is important to shed light on the temporal dynamics between pain and LTPA in daily life.

### Research questions and hypotheses

Based on the BHBM (Kolodziejczak‐Krupp et al., 2025; Wilhelm et al., 2025), this study integrates theoretical perspectives from pain psychology (e.g. fear‐avoidance model; Vlaeyen et al., 1995) and health psychology (Schwarzer & Renner, 2000) to comprehensively address LBP and back health in daily life. The overall aims of this observational study are (1) to shed more light on how LBP and back health unfold in everyday life in individuals with and without cLBP (i.e. momentary fluctuations) and to examine the extent to which these concepts are interrelated and (2) to examine how modifiable psychological risk factors (i.e. fear of movement) and resources (i.e. pain self‐efficacy), as well as health behavior (i.e. LTPA) are related to pain intensity and back health at the within‐person level. To address these aims, we implemented ecological momentary assessment (i.e. EMA), a microlongitudinal repeated measures design over several days.

We hypothesized that higher than usual momentary fear of movement, lower momentary pain self‐efficacy, and lower momentary LTPA will be associated with higher pain intensity and poorer back health. Based on the literature seeking to identify the mechanisms underlying the relationship between psychological factors and pain (e.g. Hasenbring et al., 2014; Vlaeyen et al., 1995), LTPA as a health behavior has been suggested as a potential mediating mechanism between psychological factors and LBP in previous theoretical models (Kolodziejczak‐Krupp et al., 2025). Therefore, we aimed to explore LTPA as a potential mediator at the within‐person level.

## METHODS

This study is a primary analysis of the PRIA study, a longitudinal observational study with an EMA phase (Kolodziejczak‐Krupp et al., 2025). The study is part of an interdisciplinary research consortium (FOR 5177: The Dynamics of the Spine: Mechanics, Morphology and Motion: Towards a Comprehensive Diagnosis of Low Back Pain) that aims to improve the understanding and diagnosis of cLBP and to advance its prevention and management.

### Ethical approval, funding, and transparency

The Ethics Committee of the MSB Medical School Berlin approved the study on 03/08/2021 (approval number MSB‐2021/59; amendment approved on 11/10/2023, MSB‐2023/145). The study was funded by the German Research Foundation (DFG, project number 439742772, grant numbers FL 879/2‐1, PU 762/1‐1, SCHM 2572/13‐1, and STE 477/22‐1) and was preregistered in the German Clinical Trials Register (DRKS00032978), which is also available on the International Clinical Trials Registry Platform. We report study details according to the STROBE checklist for observational studies (von Elm et al., 2008) and the CREMAS for EMA designs (Liao et al., 2016).

### Participants and procedure

Recruitment took place in the greater Berlin area via advertising (e.g. newsletters, posters, and flyers) and partnerships. Eligibility criteria were as follows: (1) reporting lumbopelvic pain for at least 12 weeks (i.e. participants with cLBP; von der Lippe et al., 2021), reporting intermittent lumbopelvic pain, or no lumbopelvic pain (i.e. participants without cLBP), (2) being between 18 and 64 years of age, (3) not being a professional, competitive, or top athlete, (4) not being currently pregnant, (5) not being enrolled in other ongoing clinical trials, (6) being able to hear a smartphone‐based alarm, and (7) reading in German on a 10‐in. tablet and 6‐in. smartphone. More specific eligibility criteria relevant to the collaborating groups of our research consortium are listed in the (FOR 5177: The Dynamics of the Spine: Mechanics, Morphology and Motion: Towards a Comprehensive Diagnosis of Low Back Pain) preregistration (DRKS00027907).

Participants were screened for eligibility at the Charité – Universitätsmedizin Berlin between January 2023 and July 2024. All participants provided written informed consent. Participants then underwent a baseline examination by a study physician and completed self‐administered tablet‐based questionnaires on LBP chronicity, intensity, disability, and sociodemographic data. A member of the study team (psychologist or sports scientist) then instructed the participants on how to use the Android study smartphone (Nokia 6.3) and the study app from movisens GmbH. Over the following 14 days, participants completed short questionnaires five times a day (alarm prompts at fixed times: 9 a.m., 12 p.m., 3 p.m., 6 p.m., 9 p.m.), resulting in up to 70 measurements per person on pain intensity, back health, fear of movement, pain self‐efficacy, and LTPA. To enhance the compatibility of the EMA with participants' daily routines, each questionnaire was available for 2.5 h after the alarm prompt. To reduce participant burden, the EMA items were embedded in a filter design that limited the number of items displayed at each measurement. Each EMA measurement was expected to take approximately 2 min to complete, resulting in a total of 2.5 h for the EMA study phase. For completing the EMA phase, participants were reimbursed €30 and received personalized feedback. Further details of the study procedures and sampling scheme can be found in the study protocol (Kolodziejczak‐Krupp et al., 2025).

### Measures

#### Low back pain chronicity, intensity, and disability

At baseline, *LBP chronicity* was self‐reported by participants using a single item that included the criterion of constant or almost daily LBP in the previous 12 weeks (von der Lippe et al., 2021). *LBP intensity* (i.e. Characteristic Pain Intensity, CPI; score ranging 0–100) and *LBP‐related disability* (i.e. Disability Score; score ranging 0–100) were measured using the German validated version of the Chronic Pain Grade Questionnaire (CPGQ; Klasen et al., 2004; von Korff et al., 1992), with instructions adapted to focus on LBP, with both scores ranging 0–100.

At each EMA measurement, participants were asked whether they were currently experiencing LBP or had experienced LBP since the last prompt. If participants answered *yes*, they were asked to rate their *pain intensity* since the last prompt on an 11‐point response scale ranging from 0 (*no pain*) to 10 (*pain as bad as it could be*), adapted from the CPGQ. In the evening, participants were additionally asked to rate the most intense LBP of the day using the same response scale.

#### Back health

At baseline, participants were asked to report their perceived *back health* using a single item based on the SF‐36 (Morfeld et al., 2011): “How would you rate the health of your lower back?”, using a 5‐point Likert‐type item with possible responses of 1 (*poor*), 2 (*not so good*), 3 (*good*), 4 (*very good*), and 5 (*excellent*). At each EMA measurement, participants rated how their lower back felt since the last prompt using the same single item adapted from the SF‐36.

#### Leisure‐time physical activity

At each EMA measurement, participants reported up to two *LTPA* of at least 10 min, indicating the type, start, and end time of the activity. For each type of activity reported, two study team members rated the metabolic equivalent to task (MET) using the 2024 Adult Compendium of Physical Activities (Herrmann et al., 2024). MET indicates the ratio of work (or activity) metabolic rate to resting metabolic rate. One MET is equal to the energy cost of sitting quietly (3.5 mL/kg/min; Herrmann et al., 2024). The start and end times of the activity were used to calculate the activity duration in minutes.

#### Fear of movement and pain self‐efficacy

At each EMA measurement, *fear of movement* was assessed with a single item based on the Tampa Scale of Kinesiophobia (TSK, Rusu et al., 2014): “At the moment, I'm afraid that I might injure myself if I am physically active,” rated with a Likert‐type item of 1 (*I completely disagree*), through 2 (*I rather disagree*), 3 (*I somewhat disagree*), 4 (*I somewhat agree*), 5 (*I rather agree*) to 6 (*I completely agree*). *Pain self‐efficacy* was assessed using a single item adapted from the German version of the PSEQ (Mangels et al., 2009; Nicholas, 2007): “At the moment, I can still do things that I enjoy doing, such as hobbies or leisure activity, despite pain,” scored on the same response scale.

#### Covariates

At baseline, participants reported their *age* in years and *sex* according to the legal options available in Germany: female, male, or diverse. *Body mass index* (BMI) was calculated as body weight in kilograms, divided by height in meters squared, as measured by a study physician at baseline. Time was represented by the *day of study*, which spanned 1–14, and *time of day*, which corresponded to the measurement across the day (1–5).

### Data preparation

Data cleaning on the LTPA involved (1) screening of all reported activities (*n* = 3969) in order to missing‐code work‐related activities (e.g. “work,” “sorting books at work,” “patrolling–I'm a guard”), nonspecific activities (e.g. “exercising” and “sports”), and other activities not listed in the 2024 Adult Compendium of Physical Activities (e.g. “sauna” and “fan mile”; total of work‐related, nonspecific, and other unclassified activities: *n* = 143, 3.5%); (2) reverse‐coding activity duration for activities with mixed start and end times; (3) missing‐coding double‐reported activities (twice on the same measurement or on two consecutive measurements) and activities with an unplausible time window (e.g. pilates from 10 p.m. to 9 a.m.; total *n* = 15, 0.4%); (4) recoding activity end to time of questionnaire completion if activity end was reported prospectively; (5) missing coding activity start and end if both activity start and end were reported prospectively (*n* = 33, 0.8%); (6) missing coding activities from the previous day that were reported after the first measurement on the following day (*n* = 5, 0.1%); (7) recoding activity duration between 1 and 9 min per measurement to 0 min, according to the item instruction, and above 180 min per measurement to 180 min, so as to cover a standardized time window of max. 3 h per measurement; and (8) recoding activity duration to 0, if no activity was reported, resulting in 0 indicating no activity at the given measurement. After cleaning the data, we calculated the sum of up to two activities per measurement and restricted the sum score to a max. of 3 h. Then, to quantify the total energy expenditure due to LTPA per measurement point, we weighted the activity duration by the respective MET (Herrmann et al., 2024). For the resulting variable *LTPA in MET minutes*, we truncated the outliers at +2*SD*.

Following recommendations for intensive longitudinal data (Bolger & Laurenceau, 2013), we separated the raw data on fear of movement, pain self‐efficacy, and LTPA into their between‐person (BP) and within‐person (WP) variable components. The BP variables represent the person‐specific means across all measurements and allow examination of inter‐individual differences. The WP variables consist of measurement‐specific deviations from the person‐specific means, and allow us to examine intraindividual variability in fear of movement, pain self‐efficacy, and LTPA over time. We used the R package *bmlm* (Vuorre, 2023; Vuorre & Bolger, 2018) for BP/WP centering. The person‐level variables age and BMI were grand‐mean centered.

**FIGURE 1:**
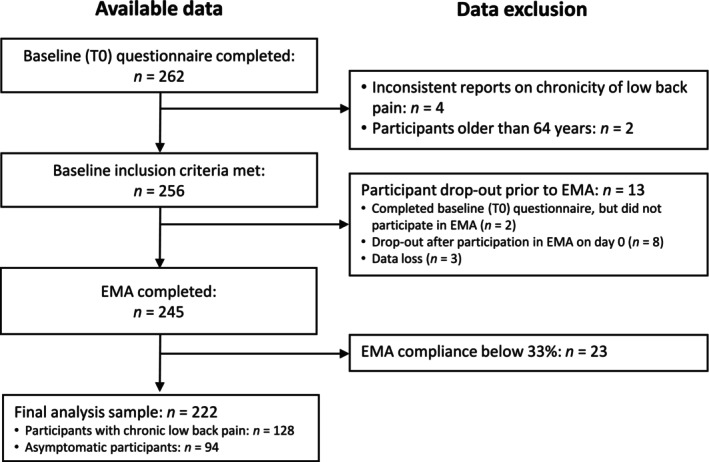
Flow diagram. Note. EMA = ecological momentary assessment.

Data on sex were coded as 1 for *female* and 0 for *male*, as no participant endorsed the response category *diverse*. Data on LBP chronicity were coded as 1 for *cLBP* and 0 for *no cLBP*. Participants were excluded from the analysis if they (1) reported cLBP during the baseline physician examination but did not report cLBP on the self‐administered questionnaire (and vice versa; *n* = 3); (2) reported cLBP with concurrent pain intensity and disability of 0 in the past 3 months (*n* = 1); (3) or were older than 64 years (*n* = 2). To ensure minimum compliance with the protocol, we excluded participants who completed less than 33% of all EMA measurements (*n* = 23; Viechtbauer, 2021). Full information on inclusion and exclusion of participants is shown in Figure 1.

### Statistical analyses

**FIGURE 2:**
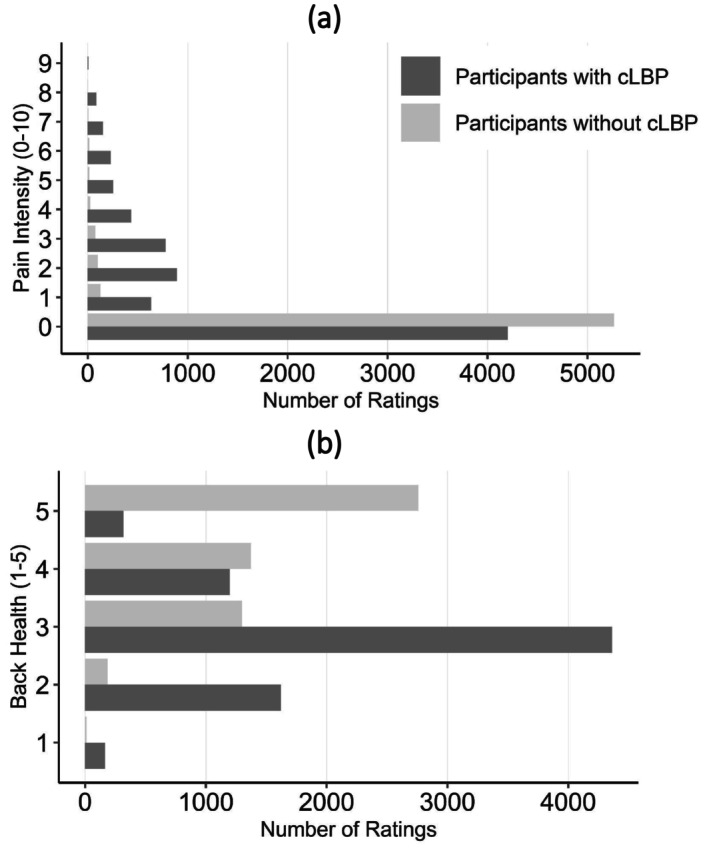
Variable distribution: pain intensity and perceived back health. Note. Each panel shows data from 13,292 measurements. Panel a presents data on pain intensity, panel b on back health. cLBP = chronic low back pain.

First, we present descriptive statistics of the baseline and daily measures for pain intensity and perceived back health. Recognizing the benefits of visualizing pain data (Lötsch & Ultsch, 2023), we plotted the raw data on momentary LBP and back health (see Figure 2). Additionally, following Song et al. (2023), we evaluated the convergent validity of our EMA measures for pain self‐efficacy and fear of movement by calculating the correlations between single‐item and multiple‐item measures of these constructs (i.e. Pain Self‐Efficacy Questionnaire [PSEQ]; Nicholas, 2007; Tampa Scale of Kinesiophobia, TSK, Rusu et al., 2014). We then estimated unconditional two‐level (measurements nested in persons) linear models with the R package *lme4* (Bates, 2014) and calculated the ICCs to determine the proportion of variance in both pain intensity and back health to be explained at the person and momentary levels. Next, with the two‐level models, we examined the associations of momentary pain intensity and back health, respectively, with fear of movement, pain self‐efficacy, and LTPA while controlling for covariates (day of study, time of day, age, sex, BMI, and LBP chronicity). As random effects, we specified random intercepts and random slopes for fear of movement, pain self‐efficacy, and LTPA. The models did not converge with *LTPA in MET minutes* or *LTPA duration in raw minutes* as predictor variables (either raw or log transformed). Thus, in the final models, we instead used the dichotomized variable *LTPA yes/no* (0 = no LTPA, 1 = engagement in LTPA since last prompt). To obtain standardized betas and confidence intervals, we z‐transformed all predictor and outcome variables prior to modeling. Associations between all momentary variables were examined using repeated measures correlations (Bakdash & Marusich, 2017) and are presented in the Supporting Information (Table S1).

To examine the theory‐based associations between fear of movement and pain self‐efficacy with LTPA, respectively, we estimated a two‐level generalized linear mixed‐effects model with the binary variable *LTPA yes/no* as the outcome variable and the psychological variables as predictors. Finally, we aimed to explore whether LTPA yes/no acts as a mediator between psychological variables and pain intensity and back health at the within‐person level, respectively (based on the BHBM; Wilhelm et al., 2025). All analyses were performed using R version 4.4.2.

## RESULTS

The flow diagram in Figure 1 shows participant inclusion. Participants (*N* = 222) were 128 individuals with cLBP and 94 individuals without cLBP, 123 women and 99 men, aged 20 to 64 years (*Mean* = 41.6, *SD* = 12.3), with BMI ranging from 17.7 to 32 (*Mean* = 24, *SD* = 3.0). Table 1 presents descriptive statistics for the variables included in the multilevel models, separately for participants with and without cLBP. Participants excluded due to completing less than 33% of all EMA measurements (*n* = 23) did not differ from the included participants on parameters relevant to cLBP at baseline (i.e. LBP chronicity, *X* ^*2*^(1, *N* = 245) = 0.01, *p* = .916; LBP intensity, *t*(243) = .15; *p* = .879, Cohen's *d* = 0.03; and LBP‐related disability, *t*(243) = −.66; *p* = .512, Cohen's *d* = 0.15).

**TABLE 1:** Descriptive statistics and intercorrelations for the variables under study.

	*M*_cLBP_	*SD*_cLBP_	*M*_no cLBP_	*SD*_no cLBP_	Intercorrelations							
					(1)	(2)	(3)	(4)	(5)	(6)	(7)	(8)
(1) Pain intensity (1–9)	3.16^a^	1.83	2.38^a^	1.51	−	−.26	.24	.15	.00	−.13	.14	−.52***
(2) Back health (1–5)	2.98^a^	0.79	4.19^a^	0.91	−.51***	−	−.17	−.06	−.11	−.01	−.43***	.29**
(3) Age (20–64)	42.5	11.7	40.5	13.0	.05	−.06	−	.02	.09	.26*	.03	.39**
(4) Female/male (in %)	57/43	−	53/47	−	.08	.02	−.04	−	−.29**	.11	−.05	−.19
(5) BMI (17.9–32.0)	23.9	3.0	24.0	2.9	.21*	−.07	.12	−.07	−	−.11	.00	.05
(6) LTPA yes/no (in %)	26/74	−	27/73	−	−.08	.00	.24**	.09	−.04	−	.29*	−.03
(7) Fear of movement (1–6)	1.85	1.16	1.89	1.32	.27**	−.25**	−.13	−.19*	−.11	−.12	−	−.23
(8) Pain self‐efficacy (1–6)	4.63^a^	1.25	5.29^a^	1.13	−.40***	.39***	−.05	.06	−.15	.03	−.39***	−

*Note*: *N* = 222 (128 participants with cLBP and 94 without cLBP). Sex is coded as 1 = female, 0 = male. Observed response range (i.e. reported minimum and maximum values) is given in brackets for each variable and may differ from the response scale. Means (*M*), standard deviations (*SD*), and intercorrelations are shown per group. Intercorrelations were calculated using baseline values (for age, sex, and BMI) and person‐specific means across all measurements (for pain intensity, back health, LTPA, fear of movement, and pain self‐efficacy) and are shown for participants with cLBP below the diagonal and for participants without cLBP above the diagonal.

Abbreviations: BMI, body mass index; cLBP, chronic low back pain; LTPA, leisure‐time physical activity.

^a^ Superscript indicates a difference in mean between the participants with cLBP and those without cLBP (tested using the *t*‐test at the *p* < .05 level).

* *p* < .05.

** *p* < .01.

*** *p* < .001.

Participants completed, on average, 59.9 of 70 EMA study questionnaires (*SD* = 11.1, range: 25–70). Out of 15,540 possible measurements, 13,292 questionnaires were completed (missing rate: 14.5%). In terms of attrition, all participants provided data for at least one measurement point up to Day 11. On Day 12, two participants did not complete any questionnaires; on Day 13, five participants; and on Day 14, 11 participants. Participants took, on average, 1.5 min to complete each questionnaire (*Median* = 1.2) and responded, on average, 34.9 min after the alarm prompt (*Median* = 10.5).

Single‐item and multiple‐item measures for pain self‐efficacy were correlated at *r* = .47, *p* < .001 for pain self‐efficacy (full sample; in participants with cLBP at *r* = .58, *p* < .001), and for fear of movement at *r* = .44, *p* < .001 (full sample; in participants with cLBP at *r* = .49, *p* < .001). Unconditional multilevel models (i.e. ICC) showed that 62% of the variance in momentary pain intensity originated from the person level. For back health, 77% of the variance originated from the person level.

### Pain intensity and back health at baseline versus in daily life

At baseline, participants with cLBP reported mean LBP intensity (CPI) of 38.7 (*SD* = 15.8, range: 6.7–80), mean LBP‐related disability (Disability Score) of 20.6 (*SD* = 20.3, range: 0–93.3), and mean back health of 2.11 (*SD* = 0.6, range: 1–4). Participants without cLBP reported mean LBP intensity (CPI) of 10.5 (*SD* = 11.4, range: 0–46.7), mean LBP‐related disability (Disability Score) of 2.06 (*SD* = 5.1, range: 0–23.3), and mean back health of 3.34 (*SD* = 0.9, range: 1–5). Mean differences between participants with and without cLBP were significant and of large size (Cohen's *d* of 2.1, 1.25, and −1.6, respectively) for all three variables.

### Fluctuations in pain intensity and back health in daily life

For the EMA measurements, the occurrence of LBP since the last prompt was reported in 3824 measurements, with participants with cLBP reporting pain in 45% of measurements (*n* = 3461), and participants without cLBP in 6.9% of measurements (*n* = 363). When pain did occur, participants with cLBP had a higher mean pain intensity than those without cLBP (*d* = 0.5; see Table 1). Participants with cLBP also reported poorer mean back health than those without cLBP (*d* = −1.4; Table 1). The repeated measures correlation for pain intensity and back health was *r* = −.62 for participants with cLBP and *r* = −.41 without cLBP, both *p*s < .001 (Table S1). Figure 2 shows the distribution of momentary pain intensity and back health for participants with and without cLBP.

**FIGURE 3:**
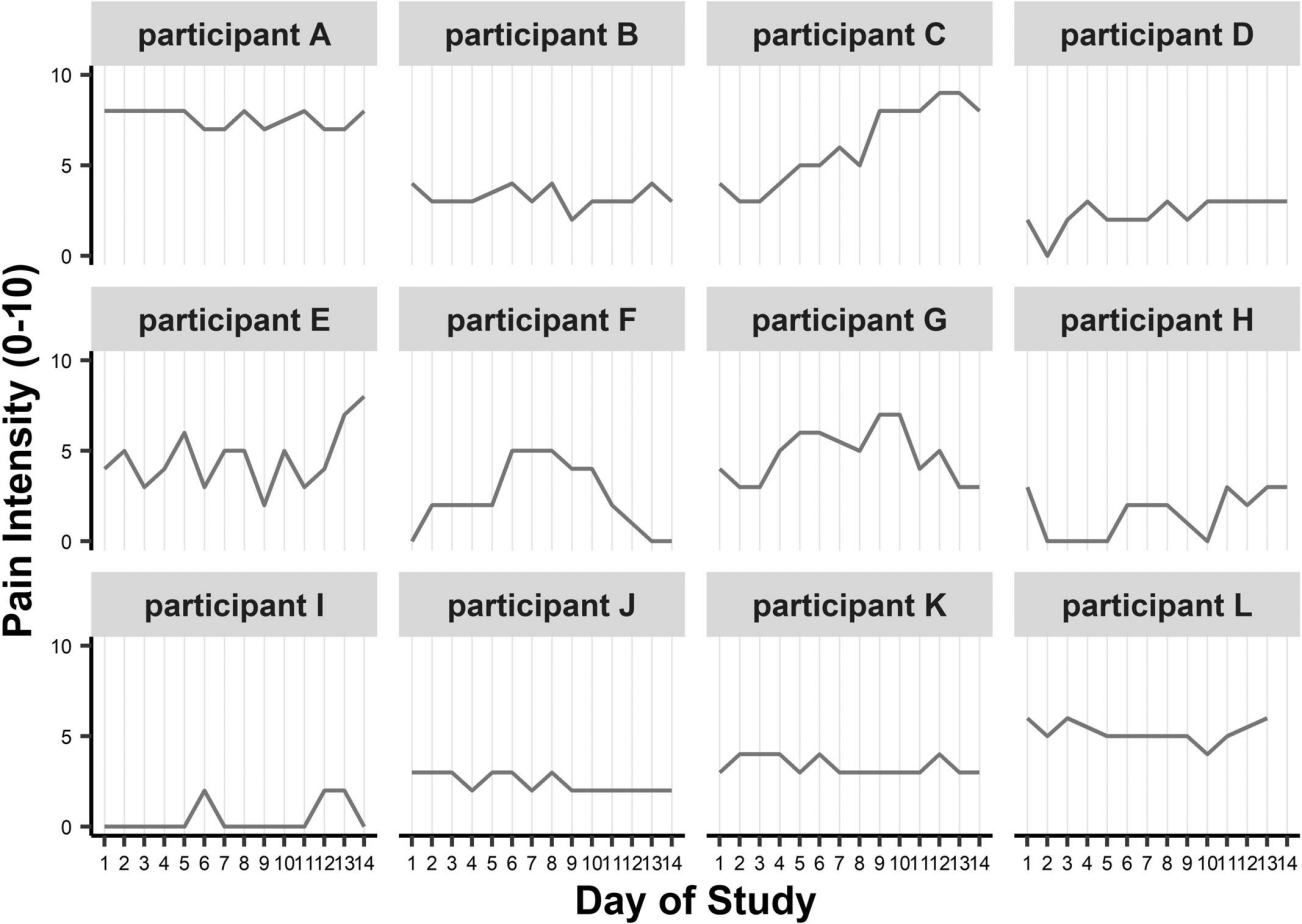
Most intense low back pain during the day across 14 days in participants with cLBP. Note. Randomly selected participants who reported cLBP at baseline. cLBP = chronic low back pain.

Among participants with cLBP, two thirds (*n* = 82) reported pain every day, whereas one third (*n* = 46) reported no LBP on at least one study day. For all participants with cLBP, the most intense LBP of the day averaged *M* = 2.67 (*SD* = 1.97, ranging 0–9) across days (for examples of daily variations in highest pain intensity, see Figure 3). Results from multilevel linear models based on 3823 measurements (Table 2) for day of study as a covariate showed that pain intensity decreased, and back health increased over the course of the day. Back health increased significantly over the study period.

**TABLE 2:** Multilevel linear models examining low back pain intensity and perceived back health, each as a function of fear of movement, pain self‐efficacy, leisure‐time physical activity, and covariates.

	Pain intensity				Back health			
	*β*	*SE*	CI	*p*	*β*	*SE*	CI	*p*
Fixed effects								
Intercept	−0.07	0.05	−0.17 to 0.04	**<.001**	0.02	0.06	−0.10 to 0.13	**<.001**
Day of study	0.00	0.01	−0.01 to 0.02	.627	0.05	0.01	0.02–0.07	**<.001**
Time of day	−0.03	0.01	−0.05 to −0.01	.**001**	0.04	0.01	0.02–0.06	.**001**
Age	0.03	0.05	−0.06 to 0.13	.500	0.09	0.06	−0.03 to 0.20	.133
Sex	0.09	0.05	−0.00 to 0.19	.052	−0.12	0.06	−0.23 to −0.02	.**026**
BMI	0.10	0.05	0.00–0.20	.**044**	−0.07	0.06	−0.19 to 0.04	.213
LBP chronicity	0.06	0.03	−0.01 to 0.12	.084	−0.07	0.04	−0.14 to 0.01	.078
LTPA (BP)	−0.09	0.05	−0.19 to 0.01	.087	0.09	0.06	−0.02 to 0.21	.103
LTPA (WP)	0.04	0.01	0.01–0.06	.**001**	−0.01	0.01	−0.03 to 0.02	.661
Fear of movement (BP)	0.09	0.06	−0.02 to 0.20	.107	−0.09	0.06	−0.21 to 0.04	.159
Fear of movement (WP)	0.05	0.02	0.00–0.09	.**042**	−0.07	0.02	−0.12 to −0.02	.**004**
Pain self‐efficacy (BP)	−0.32	0.06	−0.43 to −0.21	**<.001**	0.29	0.06	0.16–0.42	**<.001**
Pain self‐efficacy (WP)	−0.15	0.02	−0.18 to −0.11	**<.001**	0.16	0.03	0.11–0.21	**<.001**

Random effects					Random effects			
*σ* ^2^			0.25		*σ* ^2^			0.20
*τ*_00 ID_			0.37		*τ*_00 ID_			0.20
*τ*_11 ID LTPA (WP)_			0.00		*τ*_11 ID LTPA (WP)_			0.00
*τ*_11 ID Fear of movement (WP)_			0.03		*τ*_11 ID Fear of movement (WP)_			0.01
*τ*_11 ID Pain self‐efficacy (WP)_			0.02		*τ*_11 ID Pain self‐efficacy (WP)_			0.02
*ρ*_01_			.15		*ρ*_01_			−.11
			−.18					−.27
			.08					.07
Marginal *R* ^2^/conditional *R* ^2^			.239/.718		Marginal *R* ^2^/conditional *R* ^2^			.176/.622

*Note*: *N* = 3823 measurements from 183 participants. Sex is coded as 1 = female, 0 = male. Statistically significant effects (*p* < .05) are highlighted in bold.

Abbreviations: BMI, body mass index; BP, between‐person variable; CI, 95% confidence interval described by the lower limit and the upper limit; LBP, low back pain; LTPA, leisure‐time physical activity; WP, within‐person variable.

### Psychological risk factors and resources for pain intensity and back health

**FIGURE 4:**
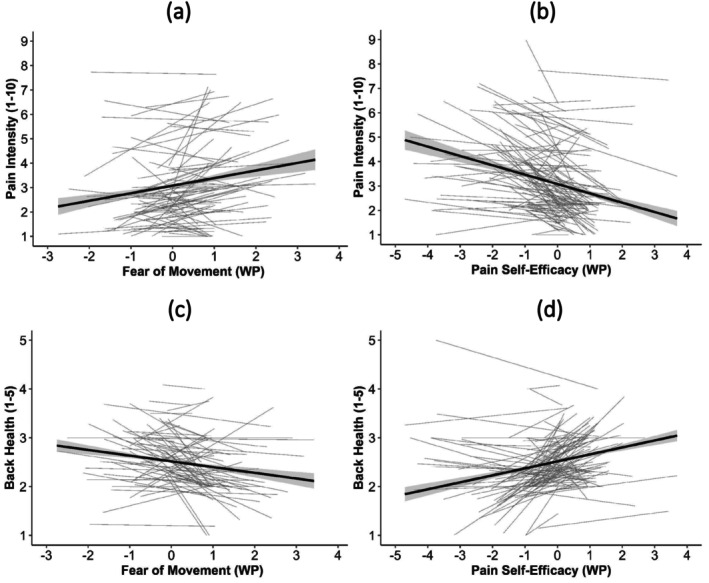
Association between the momentary outcome variables pain intensity and perceived back health and the within‐person variables fear of movement and pain self‐efficacy. Note. Each panel shows data from 3823 measurements. Panels a and b present the associations for pain intensity, panels c and d for back health. WP = within‐person variable.

The results of the multilevel linear models (Table 2) showed that at times when participants reported more fear of movement than usual, they also reported higher levels of pain intensity and poorer back health (see Figure 4). In contrast, average levels of fear of movement were unrelated to pain intensity and back health. In moments when participants experienced higher levels of pain self‐efficacy, they also experienced lower levels of pain intensity and better back health (Figure 4). Also, higher average levels of pain self‐efficacy were related to lower pain intensity and better back health.

Regarding the covariates, higher BMI was associated with higher pain intensity, and women reported poorer back health than men (Table 2). Fixed effects explained ≈24% of the variability in pain intensity and 18% in back health, and fixed and random effects together explained 72% of the variability in pain intensity and 62% in back health.

As sensitivity analyses, we included in the models interaction effects of LBP chronicity with each of the BP and WP variables. The pattern of results remained identical, with no interactions with LBP chronicity being significant, either when all interaction effects were considered simultaneously or when considered stepwise.

### Health behavior and its links to pain intensity and back health

Participants reported LTPA in 26%–27% of measurements (in participants with and without cLBP, respectively), indicating an average of once per day. When LTPA was reported, the mean LTPA intensity was 4.32 MET (*SD* = 1.6, range: 1.5–15.5) and the mean LTPA duration was 59.7 min (*SD* = 44.4, range: 10–180). Across all measurements, the mean total energy expenditure during LTPA (i.e. MET minutes) was 58.8 (*Median* = 0, *SD* = 119.2, range: 0–425.5), and the mean LTPA duration was 15.3 min (*Median* = 0, *SD* = 34.5, range: 0–180).

Results from the multilevel linear models (Table 2) revealed that at times when participants reported engaging in LTPA since the last prompt (LTPA as a binary predictor), they also reported higher pain intensity but not poorer back health. Yet, there were no associations of LTPA engagement with pain intensity or back health at the between‐person level.

Results of the generalized linear mixed‐effects model with LTPA as the binary outcome variable (Table 3) showed that the associations between LTPA and fear of movement or pain self‐efficacy, respectively, were not significant at both between‐person and within‐person levels. Therefore, we did not further explore LTPA as a potential mediator of the association between the psychological variables and pain intensity or back health. Results showed that later time of day, older age, and the absence of cLBP were related to a higher likelihood of LTPA. Fixed effects explained ≈3% of the variability in LTPA, and fixed and random effects together explained 25% of the variability in LTPA.

**TABLE 3:** Generalized linear mixed‐effects model examining leisure‐time physical activity as a function of fear of movement, pain self‐efficacy, and covariates.

Predictors	Leisure‐time physical activity (yes/no)		
	Odds ratio	CI	*p*
Intercept	0.51	0.30–0.87	.**013**
Day of study	0.99	0.97–1.01	.348
Time of day	1.10	1.04–1.16	**<.001**
Age	1.02	1.01–1.04	.**005**
Sex	1.04	0.71–1.51	.849
BMI	0.99	0.93–1.05	.648
LBP chronicity	0.47	0.30–0.76	.**002**
Fear of movement (BP)	0.95	0.78–1.17	.643
Fear of movement (WP)	1.08	0.90–1.31	.398
Pain self‐efficacy (BP)	0.98	0.79–1.23	.875
Pain self‐efficacy (WP)	1.06	0.92–1.22	.448

Random effects	
*σ* ^2^	3.29
*τ*_00 ID_	0.94
*τ*_11 ID Fear of movement (WP)_	0.04
*τ*_11 ID Pain self‐efficacy (WP)_	0.04
*ρ*_01_	.44
	−.30
Marginal *R* ^2^/conditional *R* ^2^	.031/.251

*Note*: *N* = 3823 measurements from 183 participants. Statistically significant effects (*p* < .05) are highlighted in bold.

Abbreviations: BMI, body mass index; BP, between‐person variable; CI, 95% confidence interval described by the lower limit and the upper limit; LBP, low back pain; WP, within‐person variable.

Follow‐up analyses aiming at disentangling different intensities of LTPA showed that participants reported moderate‐to‐vigorous physical activity (i.e. MVPA) on 3228 occasions (24.3% of all valid measurements), but light physical activity (i.e. LPA) on only 148 occasions (1.1% measurements). Multilevel models including MVPA as an independent variable instead of LTPA on both the between‐person (BP) and within‐person (WP) levels showed that at times when participants reported more MVPA than usual, they also reported higher pain intensity (see Table S2).

## DISCUSSION

This intensive longitudinal observational study aimed to shed light on how pain intensity and perceived back health fluctuate in the daily life of individuals with and without cLBP, and how this is shaped by psychological risk factors and resources, as well as health behavior. As expected, participants with cLBP experienced low back pain much more frequently and at a higher intensity than participants without cLBP. Participants with cLBP also reported poorer perceived back health than participants without cLBP. As hypothesized, greater fear of movement and lower pain self‐efficacy were associated with higher momentary pain intensity and poorer back health. Contrary to expectations, higher than usual engagement in LTPA was related to higher momentary pain intensity.

### Fluctuations in pain intensity and back health in daily life

As expected, pain intensity varied both between and within individuals with cLBP, and less so in people without cLBP. As shown in Figure 3, day‐to‐day fluctuations in pain intensity varied considerably between people with cLBP, including differences in mean levels, direction of change, and degree of variability. This highlights the heterogeneity of the population of people with cLBP. Importantly, several participants with cLBP experienced days with no pain or with pain of very low intensity. This again shows that, in the present sample, pain intensity fluctuates substantially in daily life and may suggest that conceptualizing chronicity as persisting *daily* or *almost daily* for at least 3 months may be at odds with the lived reality of people with cLBP.

In terms of measure validity, the EMA measures of pain intensity and perceived back health discriminated between participants with and without cLBP as well as established baseline measures (as indicated by significant mean differences), highlighting the validity and utility of such measures (Overton et al., 2023). Pain occurrence at almost half of the measurements (45%) in participants with cLBP suggests that intensive microlongitudinal designs are well suited to studying cLBP, as they have the potential to disentangle moments of no pain from moments of any pain intensity and to investigate underlying mechanisms without leading to measurement reactivity (e.g. systematic increases or decreases in reported pain intensity over time). The individualized, fine‐grained picture of cLBP may also be of clinical utility, which should be further explored in interventional studies. Importantly, in line with previous work (Gruszka et al., 2019), our results suggest that self‐reported pain intensity and perceived back health are two distinguishable constructs that capture different domains to some degree, particularly for individuals with cLBP. Specifically, different proportions of the variance in momentary pain intensity and momentary back health originated from the person level (see the ICC; 62% and 77%, respectively). Furthermore, multilevel models incorporating the same set of predictor variables explained larger proportions of the variance in momentary pain intensity than in momentary back health (*R* ^2^ of 24% and 18% for fixed effects, respectively, and 72% and 62% for fixed and random effects, respectively). Finally, repeated measures correlation for momentary pain intensity and momentary back health was *r* = −.62 for participants with cLBP and *r* = −.41 without cLBP, both *p*s < .001. This reinforces the idea that an absence of pain does not necessarily equate to a high level of subjective back health. Back health is therefore a promising patient‐reported outcome measure in cLBP research, particularly for primary and secondary prevention. Future research should aim to deepen the understanding and widen the scope of back health measurement. For example, the relationship between different aspects of back health, such as back posture, movement, and mobility (Wilhelm et al., 2025) and performance‐based measures indicative of LBP could be explored.

### Psychological risk factors and resources for low back pain and back health

Similar to previous research on factors important in the prevention and management of cLBP (Banerjee et al., 2022; Levenig et al., 2024), we examined psychological factors related to LBP. As hypothesized, greater fear of movement and lower pain self‐efficacy were related to higher pain intensity and poorer back health. The within‐person associations suggest that fear of movement and pain self‐efficacy constitute important risk factors and resources, respectively, for pain intensity and back health in everyday life. From a theoretical perspective, fear of movement contributes to the chronification of pain, especially when it persists despite healing or when it overgeneralizes (Vlaeyen et al., 2016). Importantly, fear‐avoidance beliefs have been shown to be a modifiable factor associated with cLBP (George et al., 2003; Jellema et al., 2006). Previous research has shown that greater fear‐avoidance beliefs are associated with higher pain intensity and pain‐related disability (Taheri et al., 2025; Wertli et al., 2014). Pain self‐efficacy has also been shown to be a modifiable factor and a promising coping resource (Costa et al., 2011; Miles et al., 2011). Considering this, the within‐person associations obtained in this study may inform the development of just‐in‐time adaptive interventions (JITAIs; Nahum‐Shani et al., 2018) targeting modifiable risk factors and resources for LBP and back health as a component of interdisciplinary multimodal pain therapy or to facilitate cLBP prevention (Maas et al., 2025). Implementing such “in‐the‐moment interventions” at a microtemporal level may drive change at a macro‐temporal level, thereby improving treatment outcomes. However, more research is needed. For instance, despite our findings of meaningful links to fear of movement and pain self‐efficacy, the high proportion of unexplained variance in both pain intensity and back health suggests that there are more important factors that differentiate between and within individuals that should be explored in future research.

### Health behavior and its links to pain intensity and back health

LTPA did not show a consistent pattern of findings with pain intensity and back health. Surprisingly, and contrary to our expectations, at times when participants reported more LTPA engagement than usual, they also reported higher momentary pain intensity. However, participants who reported more LTPA on average did not report higher pain intensity, suggesting that general engagement in LTPA may not exacerbate pain levels, despite possible temporary increases in pain following (or preceding) momentary physical activity. In addition, there were no significant associations between LTPA engagement and back health. As EMA studies typically disentangle the associations at the between‐person and within‐person levels, diverging patterns of results can be expected for these two levels (Kievit et al., 2013). Indeed, our study is not the first to demonstrate an association between elevated LTPA and pain at the momentary level (Chang et al., 2025). In addition, accumulating research indicates that physical activity may not be beneficial in cLBP per se but may depend on the type, intensity, duration, and frequency of activity (Carvalho et al., 2017; Comachio et al., 2024; Gordon & Bloxham, 2016; Tynan et al., 2024). For example, there may be a U‐shaped relationship between LTPA and pain intensity. That is, the *intensity* of LTPA may be crucial for the risk of cLBP, with both inactivity (e.g. high levels of sedentary behavior) and excessive activity (e.g. strenuous physical activity) exacerbating pain. In this context, moderate‐intensity LTPA may present the lowest risk for cLBP (Heneweer et al., 2009). This could explain why we did not detect a linear relationship between LTPA occurrence (as a binary variable, understood as inactivity vs. activity) and pain intensity at the between‐person level in the present sample. Similarly, there may be no linear association between the intensity of LTPA (e.g. in MET, understood as light‐intensity to high‐intensity LTPA) and pain intensity.

Next, the *type* of LTPA may be important for the risk of cLBP. A recent study showed that aerobic LTPA of more than 75 min combined with muscle‐strengthening LTPA 2 to 5 times per week had a protective effect against cLBP (Zhu et al., 2024), and another study showed that increasing moderate‐to‐vigorous LTPA by 20 min per day was associated with a lower risk of cLBP (Gupta et al., 2022). Nevertheless, further research is needed to gain a thorough understanding of the benefits of LTPA for people with cLBP. It should be noted that the present study examined concurrent associations between LTPA and pain intensity. Future studies could examine whether and how the timing of LTPA affects an individual's experience of LBP. In particular, examining the lagged effects of LTPA on pain intensity for different time intervals may shed more light on the temporal relationship between LTPA and pain.

In the present analyses, we focused on *pain‐related* psychological risk factors and resources as determinants of LTPA (Kolodziejczak‐Krupp et al., 2025; Wilhelm et al., 2025). This may be one of the explanations why the fixed effects in the model with LTPA as the outcome variable explained only a small amount of variance in LTPA (Table 3). As suggested by Crombez et al. (2012), examining theory‐based psychological correlates of pain should also adopt a self‐regulatory perspective, and this particularly applies to the context of volitional LTPA. In turn, according to the BHBM (Wilhelm et al., 2025), future research should examine alternative, more specific motivational and volitional predictors of LTPA based on the psychology of health behavior change (Schwarzer, 1992; Wilhelm et al., 2025) to facilitate theory and intervention development. Importantly, the relationship between physical activity and pain is complex and includes a range of cognitive, emotional, and behavioral responses (Hasenbring & Verbunt, 2010). The pain‐related variables selected for this analysis showed no significant associations with LTPA (see Table 3), suggesting that further mediation analyses with LTPA as the mediator were not warranted in this study. However, fine‐grained investigations of other possible pathways, such as avoidance–endurance responses to pain, may help explain the circumstances in which LTPA operates as a mediator between psychological factors and pain.

### Limitations and challenges to data assessment

Compared to clinical samples (Maas et al., 2025), our sample of individuals with cLBP, as defined by the common time‐based criterion of 12 weeks, reported low levels of pain intensity and pain‐related disability and moderate to good back health. This may be due to both the initial inclusion criterion of BMI < 28 and the demanding study design of the entire research consortium, which may have biased the sample towards high‐functioning participants. In addition, our sample consists mainly of individuals with high levels of LTPA, low levels of pain intensity, and pain‐related disability despite prolonged low back pain (i.e. longer than 12 weeks of persistent pain). Therefore, our findings may not be generalizable to individuals with higher pain intensity or other characteristics of clinical populations, such as greater comorbidity or higher pain‐related medication intake compared to people without cLBP (Gore et al., 2012). Future studies should extend our measurement protocol to a sample of individuals with more severe psychosocial and functional impairments (i.e. higher pain grade; in line with the broader clinical definition of chronicity; Treede et al., 2019), with the additional aim of refining definitions of clinically relevant pain intensities (e.g. Olsen et al., 2018). Relatedly, pain *interference* may be even more important than pain intensity as an indicator of pain‐related disability in terms of the impact of cLBP on daily life (May et al., 2018; Overton et al., 2023; von Korff et al., 2020). To further strengthen the empirical evidence, future EMA studies on pain should aim to extend our findings by additionally examining momentary pain interference and how it relates to psychological factors.

Our single‐item measures of pain self‐efficacy and fear of movement showed moderate correlations with the multiple‐item measures (and a large correlation observed in participants with cLBP for pain self‐efficacy). These findings are consistent with those reported by Song et al. (2023), where the correlations ranged from small to large‐sized. This demonstrates sufficient overlap between the single‐item and multiple‐item measures to validate the former while offering the measurement efficacy.

Self‐reports on LTPA required thorough cleaning (see Section 2.4). Self‐reported LTPA may not provide a complete picture of people's leisure‐time musculoskeletal activity, as it is limited by participants' awareness, recall, and willingness to report. Finally, our models with continuous LTPA (i.e. LTPA duration *or* MET minutes) did not converge. Due to our multiple daily assessments, the scores were all positively skewed (i.e. high proportion of 0 s), which strained the estimation procedures for linear models. Alternative methodological approaches that allow for more zeros in the data distribution (Green, 2021) or use continuous data on physical activity (e.g. accelerometry; Gupta et al., 2022) could help to address this issue in future research.

## CONCLUSION

To gain a more comprehensive picture of LBP, we need assessment methods that investigate pain experience and physical activity in the natural environment. The implementation of microlongitudinal research designs with repeated sampling of momentary experiences in daily life helps to identify potential theory‐based, modifiable targets for effective interventions. This study provided insights into daily fluctuations in pain intensity and perceived back health and their psychological and behavioral correlates in individuals with and without cLBP. Higher momentary fear of movement and lower momentary pain self‐efficacy were associated with higher pain intensity and poorer back health, whereas higher momentary LTPA engagement was associated with higher pain intensity. The within‐person associations obtained may inform the development of just‐in‐time adaptive interventions targeting modifiable risk factors and resources as well as health behaviors in individuals with cLBP.

## CONFLICT OF INTEREST STATEMENT

Karolina Kolodziejczak‐Krupp's position as research associate at MSB Medical School Berlin, Berlin, Germany, is funded by Lena Fleig's and Thomas Schäfer's grant from the German Research Foundation (grant number: FL 879/2‐1). Lena Fleig, Thomas Schäfer, Hendrik Schmidt, Matthias Pumberger, and Christoph Stein received funding from the German Research Foundation (grant numbers: FL 879/2‐1, PU 762/1‐1, SCHM 2572/13‐1, and STE 477/22‐1). The authors have no other competing interests to declare.

## ETHICS STATEMENT

The Ethics Committee of the MSB Medical School Berlin approved the study on 03/08/2021 (approval number MSB‐2021/59; amendment approved on 11/10/2023, MSB‐2023/145).

## PERMISSION TO REPRODUCE MATERIAL FROM OTHER SOURCES

The manuscript does not include any materials from sources that require permissions. All figures were generated using the R software.

## CLINICAL TRIAL REGISTRATION

The study has been registered in the German Clinical Trials Register (DRKS00032978). Date of registration: 22 December 2022.

## Supporting information


**Table S1** Repeated Measures Correlations for the Momentary Variables.
**Table S2** Multilevel Linear Models Examining Low Back Pain Intensity and Perceived Back Health, Each as a Function of Fear of Movement, Pain Self‐Efficacy, Moderate‐to‐Vigorous Leisure‐Time Physical Activity, and Covariates.

## Data Availability

Due to the research context of a chronic health issue, strict personal data protection is required. Study materials and anonymized data may be accessed upon request. Inquiries can be directed to Lena Fleig (lena.fleig@medicalschool-berlin.de).
