# Supplementary material for: Psychological risk factors and resources for low back pain intensity and back health in daily life: An ecological momentary assessment study
Source: Appl Psychol Health Well Being. 2025 Sep 22;17(5):e70080. doi: 10.1111/aphw.70080 (PMC12454926; doi:10.1111/aphw.70080)
Supplement: Supplementary file 1 — Table S1 Repeated Measures Correlations for the Momentary Variables. Table S2 Multilevel Linear Models Examining Low Back Pain Intensity and Perceived Back Health, Each as a Function of Fear of Movement, Pain Self‐Efficacy, Moderate‐to‐Vigorous Leisure‐Time Physical Activity, and Covariates. [file APHW-17-0-s001.docx]

**ONLINE SUPPLEMENTARY MATERIAL**

**Psychological risk factors and resources for low back pain and back health in daily life:**

**An Ecological Momentary Assessment study**

Karolina Kolodziejczak-Krupp^1^, Lea O. Wilhelm^1,2^, Lotte-Eleonora Diering^1,2^,

Valerie Zipper^1^, Jana Maas^3,4^, Thomas Schäfer^5^, Matthias Pumberger^6^,

Hendrik Schmidt^7^, Christoph Stein^8^, & Lena Fleig^1^

^1^Department of Psychology, MSB Medical School Berlin, Berlin, Germany,

^2^Department of Education and Psychology, Freie Universität Berlin, Berlin, Germany

^3^Institute of Medical Sociology, Charité – Universitätsmedizin Berlin, Berlin, Germany

^4^Sana Hospital Lichtenberg, Berlin, Germany

^5^Department of Psychology, HMU Health and Medical University Erfurt, Erfurt, Germany

^6^Center for Musculoskeletal Surgery, Charité – Universitätsmedizin Berlin, Berlin, Germany

^7^Julius Wolff Institute, Berlin Institute of Health at Charité – Universitätsmedizin Berlin, Berlin, Germany

^8^Department of Anaesthesiology and Intensive Care Medicine, Charité – Universitätsmedizin Berlin, Campus Benjamin Franklin, Berlin, Germany

**Table S1**
*Repeated Measures Correlations for the Momentary Variables*

|  | Intercorrelations | | | | |
| --- | --- | --- | --- | --- | --- |
|  | (1) | (2) | (3) | (4) | (5) |
| (1) Pain intensity | − | –.41^***^ | .04 | .09 | −.25^***^ |
| (2) Back health | −.62^***^ | − | −.03 | −.07 | .07 |
| (3) LTPA yes/no | −.08^***^ | −.02 | − | .01 | .00 |
| (4) Fear of movement | .16^***^ | −.12^***^ | .01 | − | −.17^***^ |
| (5) Pain self-efficacy | −.27^***^ | .22^***^ | .00 | −.17^***^ | − |

*Note*. *N* = 3,461 measurements from 128 participants with cLBP and 363 measurements from 94 participants without cLBP. LTPA = leisure-time physical activity, cLBP = chronic low back pain. Intercorrelations were calculated using repeated measures correlations (Bakdash & Marusich, 2017), and are shown for participants with cLBP below the diagonal, and for participants without cLBP above the diagonal.

^***^ *p* < .001.

**Table S2**
*Multilevel Linear Models Examining Low Back Pain Intensity and Perceived Back Health, Each as a Function of Fear of Movement, Pain Self-Efficacy, Moderate-to-Vigorous Leisure-Time Physical Activity, and Covariates*

|  | **Pain intensity** | | | | | |  |  |
| --- | --- | --- | --- | --- | --- | --- | --- | --- |
|  | β | *SE* | *CI* | | | *p* |  |  |
| **Fixed effects** |  |  |  | | |  |  | |
| Intercept | -0.06 | 0.05 | -0.17 – 0.04 | | | **<.001** |  |  |
| Day of study | 0.00 | 0.01 | -0.01 – 0.02 | | | .665 |  |  |
| Time of day | -0.03 | 0.01 | -0.05 – -0.01 | | | **.001** |  |  |
| Age | 0.03 | 0.05 | -0.07 – 0.13 | | | .542 |  |  |
| Sex | 0.09 | 0.05 | -0.00 – 0.19 | | | .060 |  |  |
| BMI | 0.10 | 0.05 | 0.00 – 0.20 | | | **.045** |  |  |
| LBP chronicity | 0.06 | 0.03 | -0.01 – 0.12 | | | .078 |  |  |
| MVPA (BP) | -0.08 | 0.05 | -0.17 – 0.02 | | | .124 |  |  |
| MVPA (WP) | 0.03 | 0.01 | 0.01 – 0.05 | | | **.004** |  |  |
| Fear of movement (BP) | 0.09 | 0.06 | -0.02 – 0.19 | | | .124 |  |  |
| Fear of movement (WP) | 0.05 | 0.02 | 0.00 – 0.09 | | | **.037** |  |  |
| Pain self-efficacy (BP) | -0.32 | 0.06 | -0.43 – -0.21 | | | **<.001** |  |  |
| Pain self-efficacy (WP) | -0.15 | 0.02 | -0.18 – -0.11 | | | **<.001** |  |  |
| **Random effects** | | | | |  |  |  |  |
| σ^2^ | 0.25 | | |  |  |  |  |  |
| τ_00_ _ID_ | 0.37 | | |  |  |  |  |  |
| τ_11_ _ID MVPA (WP)_ | 0.00 | | |  |  |  |  |  |
| τ_11_ _ID Fear of movement (WP)_ | 0.03 | | |  |  |  |  |  |
| τ_11_ _ID Pain self-efficacy (WP)_ | 0.02 | | |  |  |  |  |  |
| ρ_01_ | .14 | | |  |  |  |  |  |
|  | -.18 | | |  |  |  |  |  |
|  | .08 | | |  |  |  |  |  |
| Marginal R^2^ / Conditional R^2^ | .236 / .718 | | |  |  |  |  |  |

*Note*. *N* = 3,823 measurements from 183 participants. CI = 95% confidence interval described by the lower limit and the upper limit, BMI = body mass index, LBP = low back pain, MVPA = moderate-to-vigorous leisure-time physical activity, BP = between-person variable, WP = within-person variable. Sex is coded as 1 = female, 0 = male.
